# Antifibrotic effect of apremilast in systemic sclerosis dermal fibroblasts and bleomycin-induced mouse model

**DOI:** 10.1038/s41598-023-46737-1

**Published:** 2023-11-08

**Authors:** Tomoaki Higuchi, Kae Takagi, Akiko Tochimoto, Yuki Ichimura, Hikaru Hirose, Tatsuo Sawada, Noriyuki Shibata, Masayoshi Harigai, Yasushi Kawaguchi

**Affiliations:** 1 Division of Rheumatology, Department of Internal Medicine, Tokyo Women’s Medical University School of Medicine, Tokyo, Japan; 2 Division of Multidisciplinary Management of Rheumatic Diseases, Tokyo Women’s Medical University School of Medicine, 8-1, Kawada-cho, Shinjuku-ku, Tokyo, 162-8666 Japan; 3 Department of Dermatology, Graduate School of Medical and Dental Sciences, Tokyo Medical and Dental University, Tokyo, Japan; 4 Department of Pathology, Tokyo Women’s Medical University School of Medicine, Tokyo, Japan

**Keywords:** Drug development, Experimental models of disease, Preclinical research

## Abstract

Phosphodiesterase (PDE) 4 inhibitors have been reported to suppress the progression of dermal fibrosis in patients with systemic sclerosis (SSc); however, the precise mechanisms remain to be elucidated. Therefore, we conducted experiments focusing on the antifibrotic and anti-inflammatory effects of apremilast using dermal fibroblasts derived from patients with SSc and an SSc mouse model. Dermal fibroblasts derived from healthy controls and patients with SSc were incubated with apremilast in the presence or absence of 10 ng/ml transforming growth factor (TGF)-β1 for the measurement of intracellular cAMP levels and evaluation of mRNA and protein expression. A bleomycin-induced dermal fibrosis mouse model was used to evaluate the inhibitory effects of apremilast on the progression of dermal fibrosis. Intracellular cAMP levels were significantly reduced in dermal fibroblasts derived from patients with SSc compared with those derived from healthy controls. Apremilast reduced the mRNA expression of profibrotic markers and the protein expression of type I collagen and Cellular Communication Network Factor 2 (CCN2) in dermal fibroblasts. Additionally, apremilast inhibited the progression of dermal fibrosis in mice, partly by acting on T cells. These results suggest that apremilast may be a potential candidate for treating dermal fibrosis in SSc.

## Introduction

Systemic sclerosis (SSc) is a connective tissue disease characterized by progressive dermal fibrosis and microvascular dysregulation based on abnormal autoimmunity^1^. In the early phase of tissue fibrosis, various triggers activate the inflammatory cells. During chronic inflammation, multiple cells, including tissue-resident fibroblasts, pericytes, endothelial cells, smooth muscle cells, and epithelial cells are transformed into myofibroblasts^2^. Myofibroblasts produce excessive extracellular matrix (ECM), including type I collagen, connective tissue growth factor (CTGF), and fibronectin, in the late phase^3^. In a fibrotic environment, profibrotic molecules, such as transforming growth factor-β (TGF-β), exacerbate the vicious circle of tissue remodeling, leading to irreversible fibrosis^4^. Based on these pathophysiological findings, clinical trials of anti-inflammatory and anti-fibrotic drugs for treating dermal fibrosis in SSc have been conducted. However, most failed because of insufficient effectiveness or adverse events^5^.

Phosphodiesterases (PDEs) are hydrolases that convert circulating nucleotides into their straight form. PDEs belong to 11 families that act on cyclic adenosine monophosphate (AMP), cyclic guanine monophosphate (GMP), or both^6^. Among the PDEs, PDE4 has been particularly well studied for clinical applications^7^. PDE4 is highly expressed in the brain, cardiovascular tissues, smooth muscles, skin, and immune cells, including lymphocytes, monocytes, and macrophages^7^. PDE4 mediates G protein-coupling receptors (GPCR)/ adenylate cyclase (AC)-dependent signaling^8^. When specific ligands bind to GPCR, AC is stimulated by G-proteins and increases intracellular cAMP levels^9^. Increased intracellular cAMP activates its downstream molecules, protein kinase A (PKA), and exchange proteins directly activated by cAMP (Epac)^10^. PDE4 has four subtypes, namely PDE4A-D^7^. Recent studies have demonstrated that PDE4 inhibition regulates inflammation by modulating both innate and adaptive immune responses^11^. Based on these anti-inflammatory effects, the United States Food and Drug Administration has approved a PDE4 inhibitor, apremilast, for treating psoriatic arthritis, plaque psoriasis, and Bechet’s disease, and several clinical trials involving inflammatory diseases are ongoing^11^.

In addition to their anti-inflammatory effects, PDE4 inhibitors have anti-fibrotic effects^12^. The anti-fibrotic effect of PDE4 inhibitors has been reported in fibroblasts derived from patients with SSc and preclinical SSc animal models^13–15^. For instance, in vitro analysis showed that apremilast suppressed ECM production by SSc-derived dermal fibroblasts and TGF-β-stimulated healthy dermal fibroblasts^13^. In addition, in vivo analysis using a mouse model of dermal fibrosis showed that PDE4 inhibitors suppressed the progression of dermal fibrosis by interfering with profibrotic M2 macrophages^14^, M1 macrophages, and T cells^15^. Most of these studies suggest that the action of PDE4 inhibitors is primarily anti-inflammatory, and their anti-fibrotic effect is a consequence. Recently, evidence has accumulated that suggests the direct action of the PDE4 inhibitor on the fibrotic environment, not mediated by regulation of inflammation, such as the promising results of a phase II study of the PDE4B inhibitor BI1015550 in patients with idiopathic pulmonary fibrosis^16^.

The precise mechanisms underlying the effects of PDE4 inhibitors on SSc and their anti-inflammatory and anti-fibrotic effects still need to be fully understood. Therefore, we conducted experiments focusing on the anti-inflammatory and anti-fibrotic effects of apremilast using dermal fibroblasts derived from patients with SSc and an SSc mouse model.

## Material and methods

### Reagents

TGF-β1 was purchased from PeproTech (Rocky Hill, NJ, USA). Apremilast was purchased from AdooQ Bioscience (Irvine, CA, USA). TaqMan probes were purchased from Thermo Fisher Scientific (Waltham, MA, USA). The TaqMan probes used in our experiment were as follows: Hs00164004_m1 (*COL1A1*), Hs00164099_m1 (*COL1A2*), Hs01026927_g1 (*CTGF*), Hs00426835_g1 (*ACTA2*), and NM_002046.3 (*GAPDH*). Specific primer pairs for *PDE4A–D* were described in the Supplementary Table S1. The primary antibodies used for Western blotting were antibodies specific for type I collagen (1310-01; Southern Biotech, Birmingham, AL, USA), Cellular Communication Network Factor 2 (CCN2) (sc-14939; Santa Cruz, Santa Cruz, CA, USA), phospho-AKT (4060; Cell Signaling Technologies, Danvers, MA, USA), Akt (9272; Cell Signaling Technologies), phospho-Erk1/2 (4370; Cell Signaling Technologies), Erk1/2 (4695; Cell Signaling Technologies), GAPDH (sc-25778; Santa Cruz), phospho-Smad3 (9520; Cell Signaling Technologies), and Smad3 (9523; Cell Signaling Technologies). The primary antibodies used for immunohistochemistry were antibodies specific for alpha-smooth muscle actin (αSMA) (14968; Cell Signaling Technologies), CD3 (ab5690; Abcam, Cambridge, UK), and F4/80 (ab111101; Abcam). Bleomycin was provided by Nippon Kayaku Co., Ltd. (Tokyo, Japan).

### Dermal fibroblasts

Dermal fibroblasts derived from healthy adults were purchased from Lonza (Basel, Switzerland) and Kurabo (Osaka, Japan). Dermal fibroblasts derived from patients with SSc were obtained from the lesional areas of the forearms. Due to the lack of detailed information about the purchased dermal fibroblasts, we were unable to match such as gender and age between dermal fibroblasts derived from healthy controls and those from SSc patients. The diagnosis of SSc was made based on the 2013 American College of Rheumatology/European League Against Rheumatism classification criteria^17^. All patients had the diffuse cutaneous type within five years of non-Raynaud’s phenomenon at the time of skin biopsy was performed, and did not receive glucocorticoid or immunosuppressive treatment. Up to five passages of dermal fibroblasts from both healthy control and patients with SSc were used in this study. Fibroblasts were incubated in Dulbecco's modified Eagle’s medium (DMEM) supplemented with 10% fetal calf serum and penicillin/streptomycin at 37 °C in 5% CO_2_. The medium was replaced with serum-free DMEM 24 h before all experiments. For quantitative reverse transcription-polymerase chain reaction (RT-PCR), fibroblasts were incubated with 1–10 μM apremilast in the presence or absence of 10 ng/ml TGF-β1 for 48 h. For western blotting, fibroblasts were incubated with 10 μM apremilast in the presence or absence of 10 ng/ml TGF-β1 for 30 min to assess signal transduction, and otherwise for 72 h.

### Intracellular cAMP measurement

Intracellular cAMP levels were measured using a commercially available enzyme immunoassay (EIA) kit (Cayman Chemical, Ann Arbor, MI, USA) as previously described^18^. Briefly, 30 min before treatment, cells were treated with 3-isobutyl-1-methylxanthine to eliminate the effects of endogenous PDE activity. Thirty minutes after treatment, cell lysates were collected, and measurements were conducted according to the manufacturer’s instructions.

### RNA isolation and quantitative RT-PCR

To analyze mRNA expression in dermal fibroblasts, total RNA was isolated using a commercially available kit (Life Technologies, Carlsbad, CA, USA). An equal amount of total RNA was reverse-transcribed to synthesize cDNA. cDNA was mixed with a master mix (Thermo Fisher Scientific), and each primer was applied to a plate in triplicate. Quantitative RT-PCR was performed using TaqMan probes on a ViiA 7 Real-time PCR system (Thermo Fisher Scientific). *GAPDH* served as an internal control, and the expression of each mRNA was calculated using the comparative CT (ΔΔCT) method. The expression of *PDE4A–D* mRNA was quantified using the SYBR system. For the assessment of *PDE4A–D* expression, the PCR amplified products were electrophoresed through agarose gel stained with ethidium bromide and visualized using a transilluminator.

### Western blotting

Cultured dermal fibroblasts were washed twice with phosphate-buffered saline (PBS) twice and lysed with lysis buffer supplemented with protease inhibitors on ice. The protein concentrations were calculated using a bicinchoninic acid protein assay kit (Thermo Fisher Scientific). Equal amounts of protein were applied in a 4–20% Tris–glycine gel (Thermo Fisher Scientific) and separated by sodium dodecyl sulfate–polyacrylamide gel electrophoresis. The gels were then transferred onto polyvinylidene fluoride (PVDF) membranes. After blocking with 5% skim milk for 1 h, membranes were incubated with primary antibodies at 4 °C overnight. The membranes were washed three times with PBS and incubated with secondary antibodies. Signals were visualized using an electrochemical solution (Wako, Osaka, Japan). Band density was calculated using ImageJ software (National Institutes of Health, Bethesda, MD, USA).

### Mice

BALB/c mice were purchased from Sankyo Labo Service Co., Ltd. (Tokyo, Japan). The protocol for developing bleomycin-induced dermal fibrosis in mice has been described previously^18^. Briefly, bleomycin was dissolved in 1 mg/mL PBS and sterilized by filtration. Six-week-old female mice were shaved on their backs and injected subcutaneously with either 300 μL PBS or an equal amount of bleomycin. The injections were repeated five times a week for four weeks. Apremilast was dissolved in PBS at 1 mg/kg or 5 mg/kg and injected intraperitoneally at the same time as bleomycin injection in the apremilast treatment group. After completing the protocol, the mice were sacrificed according to the guidelines of the Ethical Review Committee of Animal Experiments, Tokyo Women’s Medical University, and the back skin was cut. The skin specimens were fixed in 10% formaldehyde and embedded in paraffin.

### Histological assessment

The embedded tissue was sectioned at 3 μm thickness (MBL, Tokyo, Japan; Sept Sapie, Tokyo, Japan). To assess the dermal thickness, the slides were stained with Masson’s trichrome stain (MBL, Tokyo, Japan; Sept Sapie, Tokyo, Japan). The distance from the epidermal-dermal junction to the dermal-fat junction was measured. Images of stained slides were scanned, and the average dermal thickness of five randomly selected fields at equal magnifications was calculated using ImageJ software. The slides were blinded when measuring the dermal thickness.

### Immunohistochemistry

For immunohistochemical analysis, the sections were deparaffinized and incubated with 3% hydrogen peroxide after antigen retrieval and blocked with 5% skim milk solution. The sections were incubated with primary antibodies overnight at 4 °C and then with secondary antibodies for 1 h. The primary antibodies used were anti-αSMA (1:200), CD3 (1:50), and F4/80 (1:200). The antibodies were visualized using the DAKO EnVision system (DAKO, Santa Clara, CA, USA). The sections were developed with 3,3′-diaminobenzidine tetrahydrochloride dihydrate and counterstained with hematoxylin. The sections were photographed at 100 × magnification. The number of positively stained cells in the three skin images was counted for each group of mice in a blinded manner. Averaged positive cell numbers were used for statistical analysis.

### Collagen content

We collected skin samples from mice using a 6 mm biopsy punch (KAI Industries, Gifu, Japan) and quantified the samples using a QuickZyme total collagen assay kit (QuickZyme Biosciences, Leiden, Netherlands), following the manufacturer's instructions.

### Statistical analysis

Data are presented as the mean ± standard deviation (SD). The Mann–Whitney *U* test was used to compare the two groups. One-way analysis of variance (ANOVA) followed by Tukey’s post-hoc test was used for multiple group comparisons. Data were analyzed using JMP statistical software (Japanese version 16, SAS Institute Inc., Cary, NC, USA). p < 0.05 was considered statistically significant.

### Ethics approval

This study was performed in accordance with the Declaration of Helsinki and relevant guidelines and regulations. This study was approved by the Ethics Committee of the Tokyo Women’s Medical University (No. 3097-R). All participants were informed of the contents of this study and written informed consent was obtained.　All animal experiments in this study were approved by the Ethical Review Committee of Animal Experiments at Tokyo Women’s Medical University (AE21-136) and conducted in compliance with the ARRIVE guidelines.

## Results

### Decreased intracellular cAMP levels were associated with the fibrogenic phenotype of SSc dermal fibroblasts

**Figure 1:**
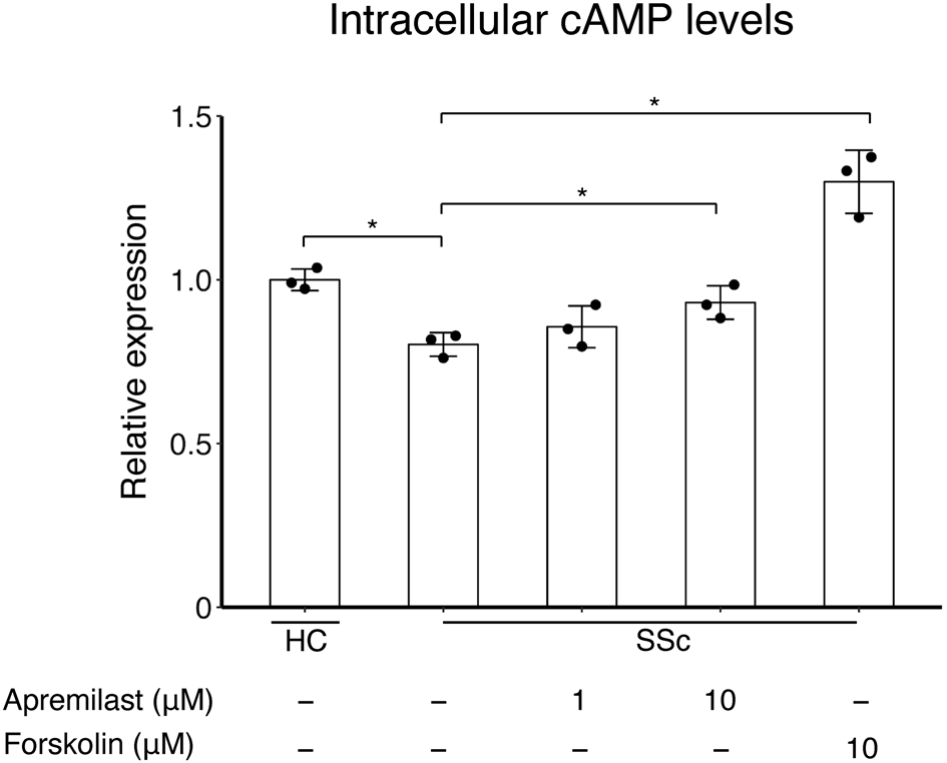
Intracellular cAMP levels in HC (n = 3) and SSc (n = 3) dermal fibroblasts in the presence or absence of 1–10 μM apremilast. The 10 μM forskolin served as a positive control. The data were expressed as the mean ± SD per group. *p < 0.05. cAMP, cyclic Adenosine 5'-Monophosphate; HC, healthy control; SD, standard deviation; SSc, systemic sclerosis.

We confirmed that dermal fibroblasts express all PDE4 subtypes (PDE4A, B, C, and D) (Supplementary Fig. S1). TGF-β1-treated dermal fibroblasts did not show increased expression of *PDE4A–D* mRNA compared to dermal fibroblasts without TGF-β1 treatment (Supplementary Fig. S2). Furthermore, there was no difference in the mRNA expression of *PDE4A–D* between healthy dermal fibroblasts and SSc dermal fibroblasts (data not shown). In our previous study, 2-carba-cyclic phosphatidic acid (2ccPA), which increases intracellular cAMP levels, decreased ECM production in SSc dermal fibroblasts in vitro and prevented the progression of dermal fibrosis in vivo experiment using a bleomycin-induced dermal fibrosis model^18^. Therefore, we hypothesized that intracellular cAMP levels are associated with fibrotic status in dermal fibroblasts and examined intracellular cAMP levels in healthy and SSc dermal fibroblasts using an EIA kit. Consistent with our previous study, intracellular cAMP levels in SSc dermal fibroblasts were significantly lower than those in healthy dermal fibroblasts (Fig. 1). Additionally, apremilast increased intracellular cAMP levels in SSc dermal fibroblasts in a dose-dependent manner similar to forskolin, a positive control to increase intracellular cAMP levels (Fig. 1). These results suggest that decreased intracellular cAMP levels in SSc dermal fibroblasts are a characteristic of the fibrotic phenotype, and that apremilast could reverse this phenotype by increasing intracellular cAMP levels in dermal fibroblasts.

### Apremilast decreased the expression of fibrotic markers in dermal fibroblasts

**Figure 2:**
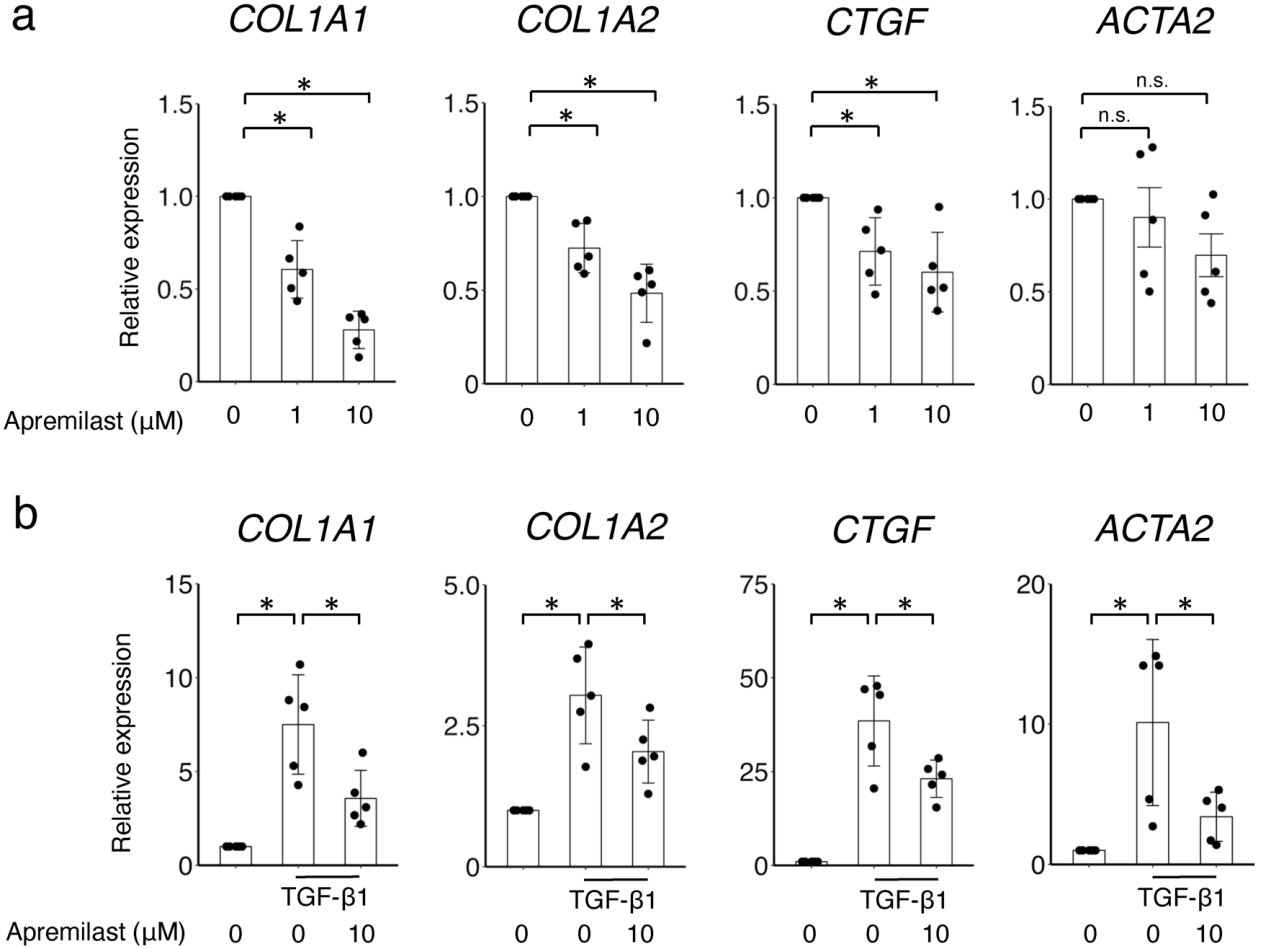
Effect of apremilast on the mRNA expression of profibrotic markers in dermal fibroblasts. (**a**) SSc dermal fibroblasts (n = 5) were incubated with 1–10 μM apremilast for 48 h, and total mRNA was collected for assessment. No treatment group served as control. (**b**) Dermal fibroblasts from HC (n = 5) were incubated with 1–10 μM apremilast in the presence or absence of 10 ng/ml TGF-β1 for 48 h, and total mRNA was collected for assessment. Results were representative of at least two independent experiments. The data were expressed as the mean ± SD per group. *p < 0.05. n.s., not significant. TGF, transforming growth factor. See Fig. 1 for other abbreviations.

**Figure 3:**
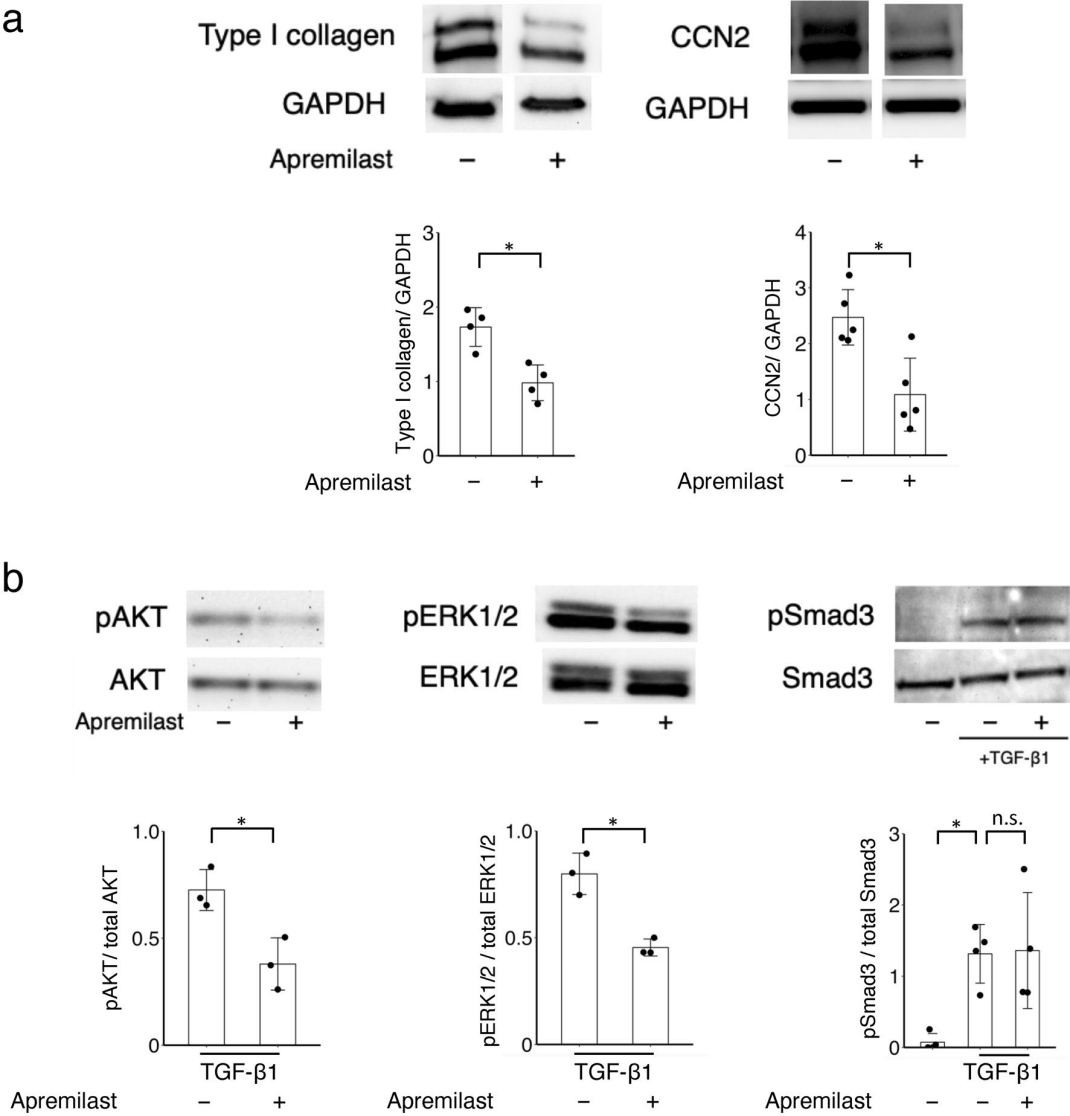
Apremilast suppressed the production of extra matrix molecules via the non-Smad pathway. (**a**) The expression of type I collagen (n = 4) and CCN2 (n = 5) in SSc dermal fibroblasts. SSc dermal fibroblasts were incubated with 10 μM apremilast for 72 h, and the cell lysate was collected for assessment. (**b**) The expression of pAkt/total Akt (n = 3), pERK1/2/total ERK1/2 (n = 3), and pSmad3/totalSmad3 (n = 4) in dermal fibroblasts. (**c**) SSc dermal fibroblasts were incubated with 10 μM apremilast for 30 min, and the cell lysate was collected for assessment. To assess pSmad3 and total Smad3, dermal fibroblasts from HC (n = 4) were incubated in the presence or absence of 10 ng/ml TGF-β1. The data were expressed as the mean ± SD per group. *p < 0.05. *n.s.* not significant. Results were representative of at least two independent experiments. CCN2 cellular communication network factor 2. See Fig. 1 for other abbreviations.

To assess the inhibitory effects of apremilast on ECM production in SSc dermal fibroblasts, SSc dermal fibroblasts were incubated with or without apremilast. Apremilast significantly reduced the expression of *COL1A1, COL1A2,* and *CTGF* mRNA in SSc dermal fibroblasts in a dose-dependent manner (Fig. 2a). We also examined the anti-fibrotic effects of apremilast in healthy dermal fibroblasts. Healthy dermal fibroblasts acquire a profibrotic, SSc-like phenotype in the presence of TGF-β1^2^. Similarly, the upregulation of ECM mRNA markers induced by TGF-β1 was significantly diminished by apremilast treatment (Fig. 2b). At the protein level, the expression of type I collagen and Cellular Communication Network Factor 2 (CCN2) in SSc fibroblasts was reduced in the presence of 10 μM apremilast (Fig. 3a). Next, we explored the downstream signaling pathways of TGF-β related to the anti-fibrotic effect of apremilast in SSc fibroblasts using western blotting. The downstream signaling pathways of TGF-β1 are classified as the canonical and non-canonical pathways; the former is known as the Smad pathway, and the latter includes the ERK1/2, p42/44, and phosphatidylinositol-3 kinase (PI3K)/Akt pathways^19^. As a result, phosphorylation of Smad3 was not suppressed by the addition of apremilast in healthy dermal fibroblasts stimulated with TGF-β1. In contrast, apremilast suppresses the phosphorylation of ERK1/2 and Akt (Fig. 3b). These results suggest that apremilast acts via several non-canonical TGF-β signaling pathways.

### Apremilast attenuated the progression of dermal fibrosis in bleomycin-induced mice model

**Figure 4:**
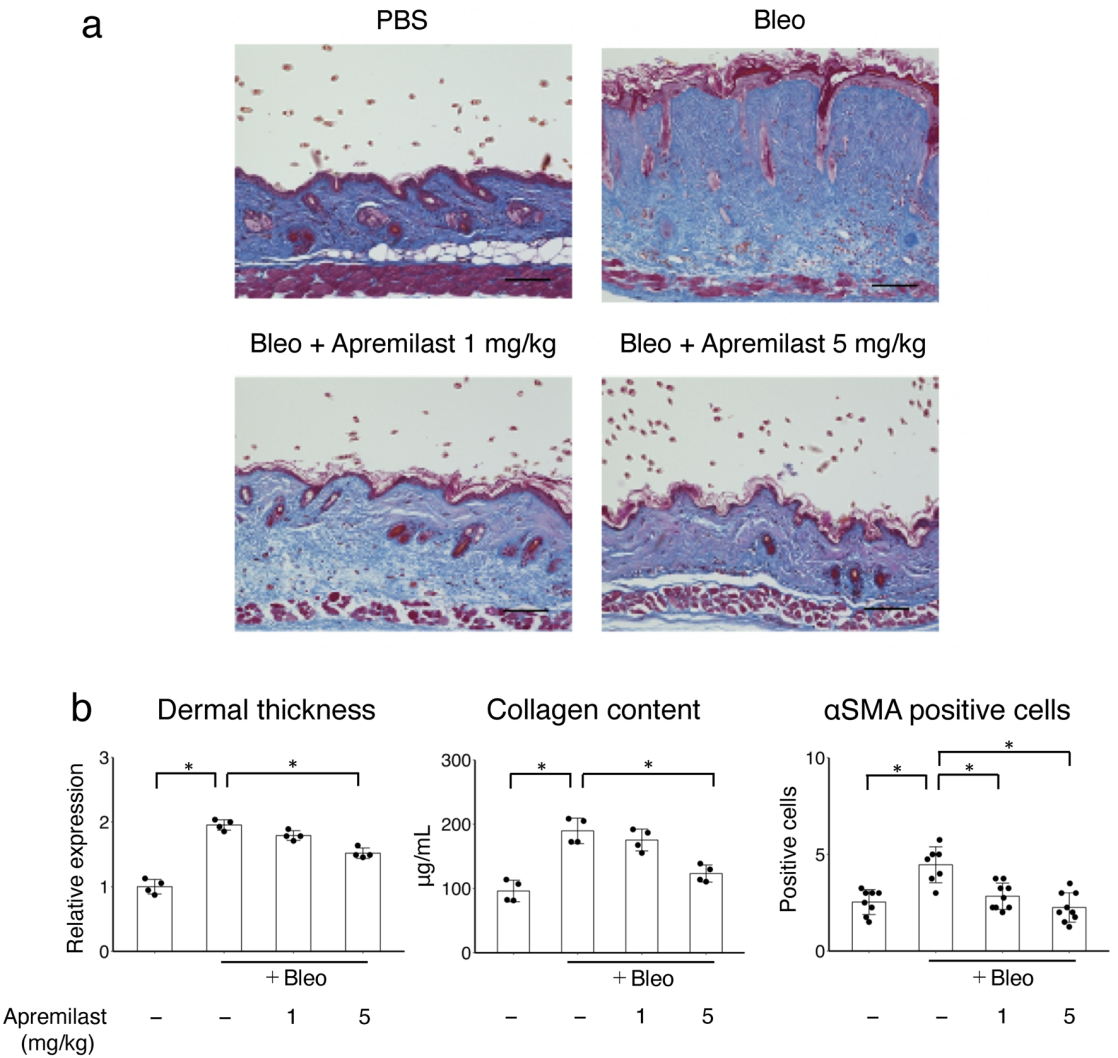
The effect of apremilast on the development of bleomycin-induced dermal fibrosis in mice. Mice were injected with either PBS or bleomycin subcutaneously on the back five times a week for four weeks. In the apremilast treatment group, 1 mg/kg/day or 5 mg/kg/day apremilast was administered intraperitoneally concomitant with bleomycin. (**a**) Representative images of Masson-Trichrome staining of the skin in mice treated with subcutaneous PBS, bleomycin, and bleomycin plus intraperitoneal injection of 1 mg/kg/day or 5 mg/kg/day apremilast. (**b**) Dermal thickness (each group, n = 4), collagen content (each group, n = 4), and the number of αSMA-positive cells (at 100 × magnification; each group, n ≥ 7) in the lesional skin. Scale bar 100 μm. *p < 0.05. Bleo bleomycin, PBS phosphate-buffered saline, αSMA alpha-smooth muscle actin. See Fig. 1 for other abbreviations.

It has been reported that bleomycin-induced dermal fibrosis in mice replicates inflammation and fibrosis in the dermal lesions of SSc^20^. We used this model to investigate the anti-fibrotic effects of apremilast in vivo. The 6-week-old BALB/c mice were injected with either PBS or bleomycin subcutaneously on the back 5 times a week and 1 mg/kg/day or 5 mg/kg/day of apremilast in PBS was intraperitoneally administered concomitantly with bleomycin. After 4-week treatment, the progression of bleomycin-induced dermal thickness was suppressed by apremilast (5 mg/kg/day) (Fig. 4). Similarly, the increased collagen content and the number of αSMA-positive cells were reduced by apremilast (5 mg/kg/day) treatment (Fig. 4).

**Figure 5:**
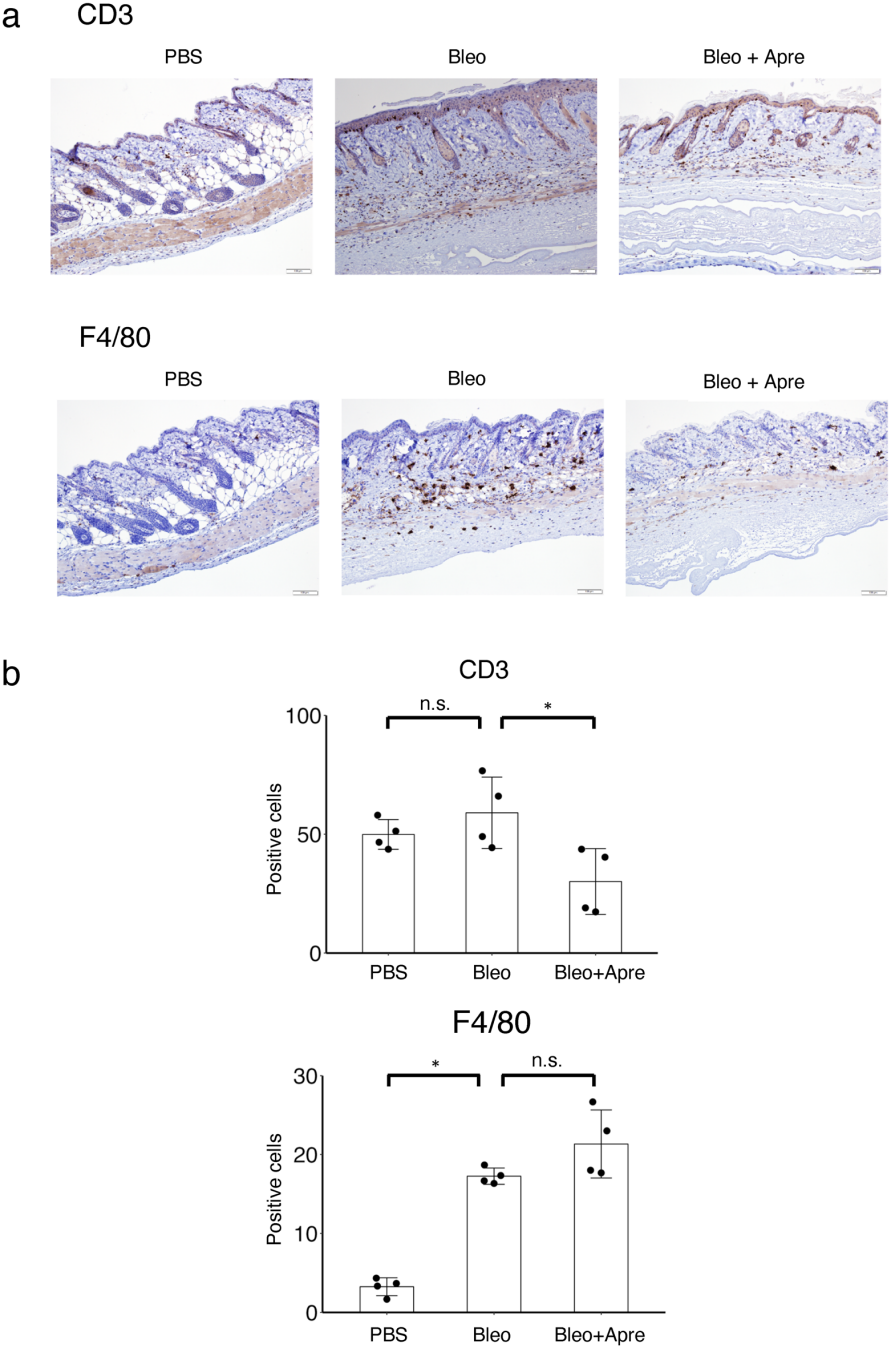
The effect of apremilast on the expression of CD3- and F4/80-positive cells in bleomycin-induced dermal fibrosis in mice. Mice (each group, n = 4) were injected with either PBS or bleomycin subcutaneously on the back five times a week for four weeks. In the apremilast treatment group, 5 mg/kg/day apremilast was administered intraperitoneally concomitant with bleomycin. (**a**) Representative images of immunohistochemical analysis with CD3 and F4/80 staining the skin in mice treated with subcutaneous PBS, bleomycin, and bleomycin plus 5 mg/kg/day apremilast. (**b**) The number of CD3 and F4/80 positive cells in the lesional skin at 100 × magnification. Scale bar 100 μm. *p < 0.05. n.s. not significant, Apre apremilast, Bleo bleomycin. See Fig. 1 for other abbreviations.

T lymphocytes and macrophages have been reported to be crucial players in the anti-inflammatory effect of apremilast^7^. Thus, we explored the effect of apremilast on T lymphocytes and macrophages by counting the number of infiltrating cells in the lesional skin. Apremilast treatment significantly decreased the number of CD3^+^ T lymphocytes, but not that of F4/80^+^ macrophages (Fig. 5).

## Discussion

In this study, we revealed that intracellular cAMP levels in SSc dermal fibroblasts were lower than those in healthy dermal fibroblasts, and that apremilast increased intracellular cAMP levels in SSc dermal fibroblasts. Furthermore, treatment with apremilast decreased ECM production in SSc dermal fibroblasts and TGF-β1-stimulated healthy dermal fibroblasts. These anti-fibrotic effects of apremilast on dermal fibroblasts may be mediated through the non-canonical downstream pathways via TGF-β, according to our in vitro analysis. Furthermore, apremilast ameliorated the progression of bleomycin-induced dermal fibroblasts in vivo partially through its action on macrophages.

Our results suggest that low intracellular cAMP levels are characteristic of profibrotic fibroblasts. The anti-fibrotic mechanisms of cAMP reported to date include inhibiting epithelial-mesenchymal transition (EMT) and inhibiting ECM synthesis by fibroblasts^13,15,21,22^. The results of our in vitro experiments were consistent with those of recent studies and suggested that the increase in cAMP levels may reverse the profibrotic phenotype of SSc dermal fibroblasts to that of normal dermal fibroblasts. Another reported profibrotic mechanism of the increase in intracellular cAMP in fibroblasts is the inhibition of cell proliferation and promotion of cell death^10^. However, this mechanism is controversial; for example, increased intracellular cAMP levels have been reported to inhibit apoptosis of gastric mucosal cells via the prostaglandin E_2_-PKA pathway^23^. Our preliminary viability assay (data not shown) and the results of previous studies indicated that cell death did not occur at apremilast concentrations up to 10 μM. Therefore, it is unlikely that apremilast induced apoptosis in fibroblasts at the concentrations we used in the present study. The effects of cAMP regulation on cell proliferation, metabolism, and cell death under fibrotic conditions require further investigation.

We demonstrated that apremilast interferes with the non-canonical TGF-β pathway. This was suggested to be the underlying molecular mechanism of the anti-fibrotic effect of apremilast. A previous report revealed that apremilast, but not Akt, interferes with the phosphorylation of Smad3 at 15 min and Erk1/2 at 30 min in TGF-β-stimulated dermal fibroblasts^13^. Contrary to the previous report, we demonstrated an association with the PI3K/Akt pathway. The PI3K/Akt pathway involves numerous biological processes such as cell proliferation, apoptosis, angiogenesis, and glucose metabolism^24^. The PI3K/Akt pathway has also been reported to be an essential signaling pathway in fibrosis^25^. Our results and a previous report suggested that the PI3K/Akt pathway is also involved in the anti-fibrotic effect of apremilast^15^, but further experiments are needed to identify specific signaling pathways. Apremilast likely suppresses multiple signaling pathways associated with TGF-β.

There is much evidence regarding the anti-inflammatory effects of apremilast. It has been reported that apremilast acts on epithelial cells, macrophages, dendritic cells, and lymphocytes^7^. PDE4 inhibition has pleiotropic immunomodulatory effects, such as decreased tumor necrosis factor (TNF) and interleukin (IL)-12 release from macrophages and dendritic cells^26^, the reduced release of IL-2 and interferon-γ from Th1 cells, IL-4 and IL-13 from Th2 cells, IL-17 and IL-22 from Th17 cells, and autoantibody production from B cells^7,11^. Previous studies on the anti-fibrotic effect of PDE4 inhibitors have focused on macrophages and T lymphocytes and have suggested that dermal fibrosis is reduced as an indirect effect of inhibitory action on these cells^14,15^. Cutolo et al*.* reported a direct effect of apremilast on fibroblasts, including SSc dermal fibroblasts^13^. However, Maier et al*.* reported that rolipram, a PDE4 inhibitor different from apremilast, neither inhibited ECM molecule production nor altered the artificial scratch closure time in both healthy and SSc dermal fibroblasts^14^. To explore the reason for discrepancy between the previous reports and the present study, we hypothesized that the inhibition of PDE7, which is not inhibited by rolipram, might also exert an anti-fibrotic effect. BRL-50481, a PDE7 inhibitor, was added to dermal fibroblasts to determine whether it had an anti-fibrotic effect on ECM mRNA expression. However, contrary to our hypothesis, BRL-50481, a PDE7 inhibitor, did not inhibit ECM mRNA expression in dermal fibroblasts (data not shown). Our results and other reports support a direct anti-fibrotic effect of apremilast on dermal fibroblasts; however, whether this effect is unique to apremilast requires further investigation.

The limitation of our study is as follows: the difference in intracellular cAMP levels between SSc dermal fibroblasts and normal dermal fibroblasts under general culture conditions may require validation with multiple datasets and larger sample sizes. We observed only numerical changes in macrophages and T lymphocytes relative to apremilast treatment, and further studies are needed to determine functional changes. While this study provides data suggesting that apremilast may inhibit fibrosis through the non-canonical TGF-β signaling pathway, a detailed analysis of all Mitogen-activated Protein Kinase (MAPK)-related pathways, including c-Jun N-terminal kinase (JNK) and p38, has not been performed. To clearly establish the direct effects on dermal fibroblasts independent of *in viv*o inflammation, further validation using such as tight skin-1 mice or mice overexpressing TGF-β receptor I is necessary.

In conclusion, our study demonstrated that apremilast inhibited the production of ECM molecules by SSc dermal fibroblasts and TGF-β1-treated healthy dermal fibroblasts in vitro. Apremilast increased intracellular cAMP levels and thus inhibited the non-canonical TGF-β pathway. Additionally, apremilast inhibited the progression of dermal fibrosis, partly through its anti-inflammatory effect on CD3^+^T lymphocytes. These results suggest that apremilast may be a potential candidate for treating dermal fibrosis in patients with SSc.

### Supplementary Information

Supplementary Information.

## Data Availability

The datasets used and analyzed during the current study are available from the corresponding author upon reasonable request.
