# Supplementary material for: Antifibrotic effect of apremilast in systemic sclerosis dermal fibroblasts and bleomycin-induced mouse model
Source: Sci Rep. 2023 Nov 8;13:19378. doi: 10.1038/s41598-023-46737-1 (PMC10632419; doi:10.1038/s41598-023-46737-1)
Supplement: Supplementary file 1 — Supplementary Information. [file 41598_2023_46737_MOESM1_ESM.docx]

**Supplementary information**

**Antifibrotic effect of apremilast in systemic sclerosis dermal fibroblasts and bleomycin-induced mouse model**

Tomoaki Higuchi ^1,2,^ *, Kae Takagi^1^, Akiko Tochimoto^1^, Yuki Ichimura^1,3^, Hikaru Hirose^1^, Tatsuo Sawada^4^, Nobuyuki Shibata^4^, Masayoshi Harigai^1^, and Yasushi Kawaguchi ^1^

^1^Division of Rheumatology, Department of Internal Medicine, Tokyo Women's Medical University School of Medicine, Tokyo, Japan

^2^Division of Multidisciplinary Management of Rheumatic Diseases, Tokyo Women's Medical University School of Medicine, Tokyo, Japan

^3^Department of Dermatology, Graduate School of Medical and Dental Sciences, Tokyo Medical and Dental University, Tokyo, Japan

^4^Department of Pathology, Tokyo Women’s Medical University School of Medicine, Tokyo, Japan

*E-mail: higuchi.tomoaki@twmu.ac.jp

**Supplementary Table 1**

| Gene | Primer sequence (5’ > 3’) | Primer bank ID | Product size (bp) |
| --- | --- | --- | --- |
| PDE4A F | ACACACCTGTCAGAAATGAGC | 341572546c1 | 99 |
| PDE4A R | GGGTGATGGGATCTCCACTTC |  |  |
| PDE4B F | AGATGAGCCGATCAGGGAAC | 82799485c2 | 101 |
| PDE4B R | CCTGTCTTTCTGGGTAGGAGA |  |  |
| PDE4C F | CAAGGCCATGTCTCGGAACTC | 341604763c1 | 243 |
| PDE4C R | AGCTCGTCTAGCGTCTCCAA |  |  |
| PDE4D F | ACGGACCGGATAATGGAGGAG | 308387383c3 | 106 |
| PDE4D R | ATTTTTCCACGGAAGCATTGTG |  |  |
| β-actin F | CATGTACGTTGCTATCCAGGC | 4501885a1 | 250 |
| β-actin R | CTCCTTAATGTCACGCACGAT |  |  |

Supplementary Table 1: Specific primer pairs for phosphodiesterase (PDE) 4A–D and β-actin. The website Primer Bank (<http://pga.mgh.harvard.edu/primerbank>) was used to design the primers for reverse-transcription polymerase chain reaction (PCR) and quantitative PCR.

**Supplementary Figure 1**


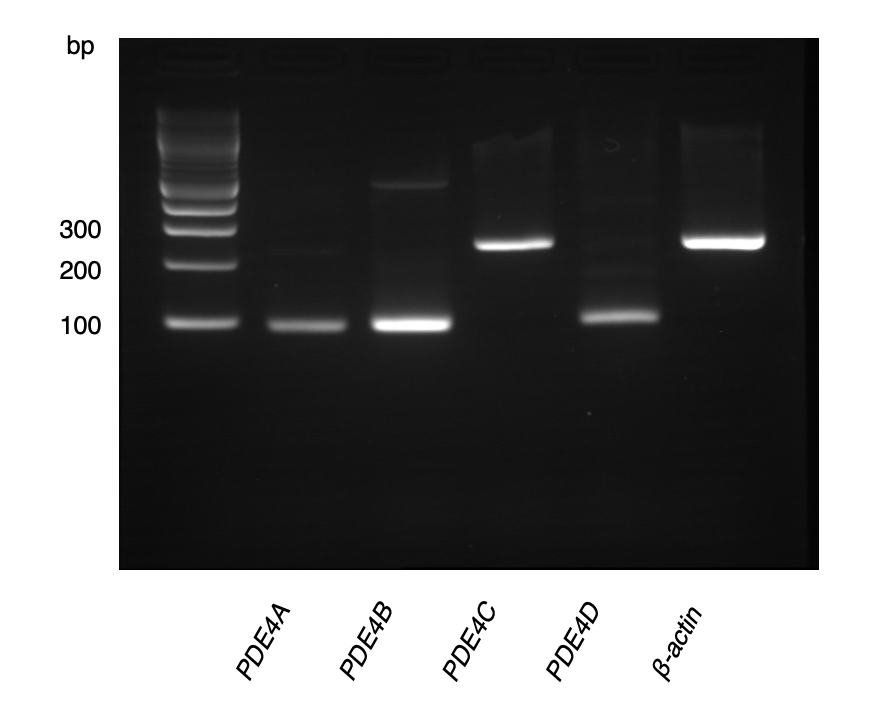


Supplementary Figure 1: The representative gel image of polymerase chain reaction amplified products of *phosphodiesterase (PDE) 4A–D* and *β-actin* in healthy dermal fibroblasts. bp, base pair.

**Supplementary Figure 2**


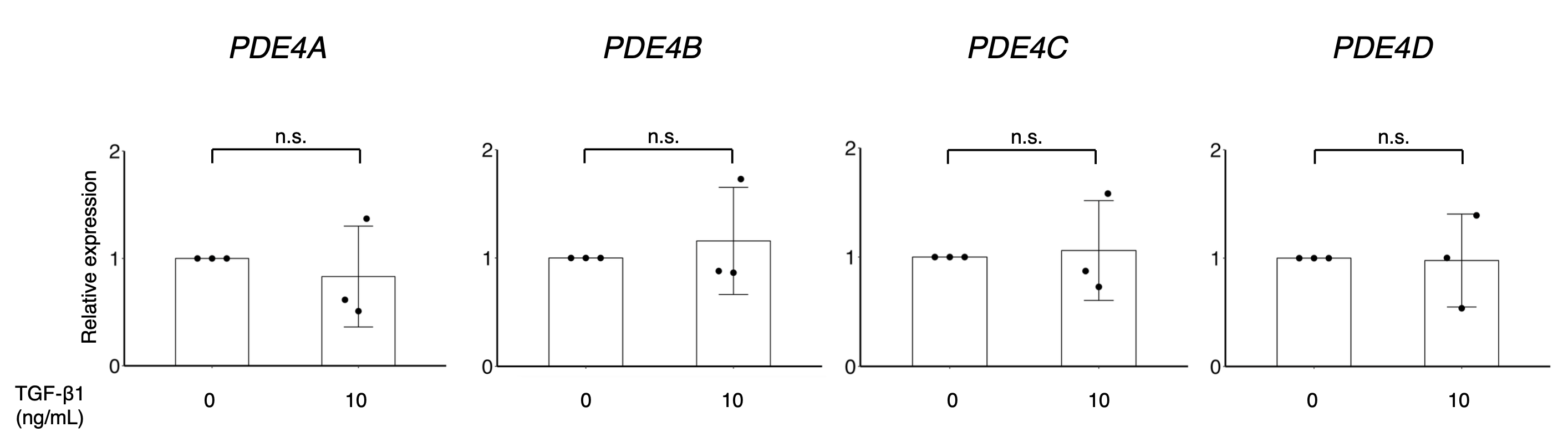


Supplementary Figure 2: Relative expression of *phosphodiesterase (PDE) 4A–D* mRNA in healthy dermal fibroblasts (n=3). Healthy dermal fibroblasts were incubated with or without TGF-β1 (10 ng/ml) for 24 h. β-actin was served as internal control. Results were representative of at least two independent experiments. The data are expressed as mean ± SD. The Mann-Whitney U test was used for statistical analysis. n.s., not significant.

**Supplementary Figure 3**


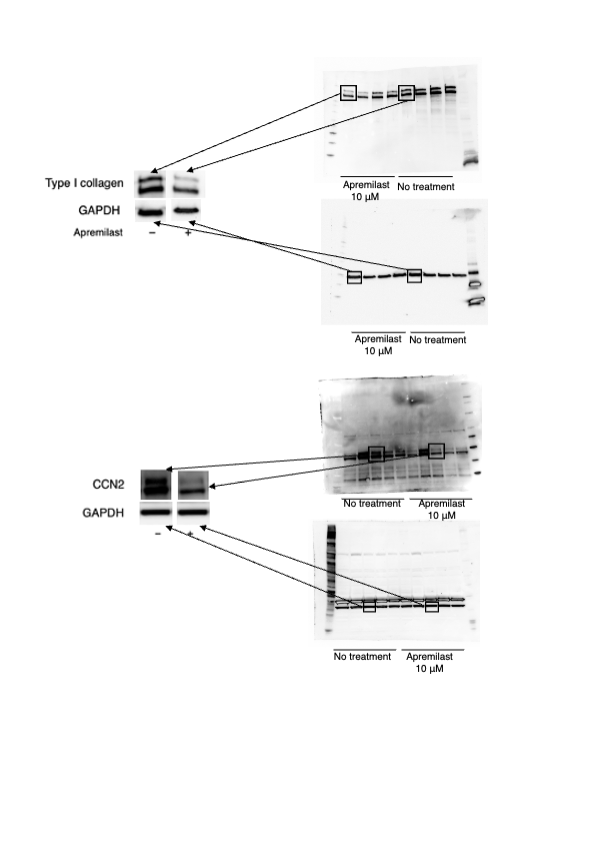


Supplementary Figure 3. Full-length images of Western blotting in Figure 2a.

**Supplementary Figure 4**


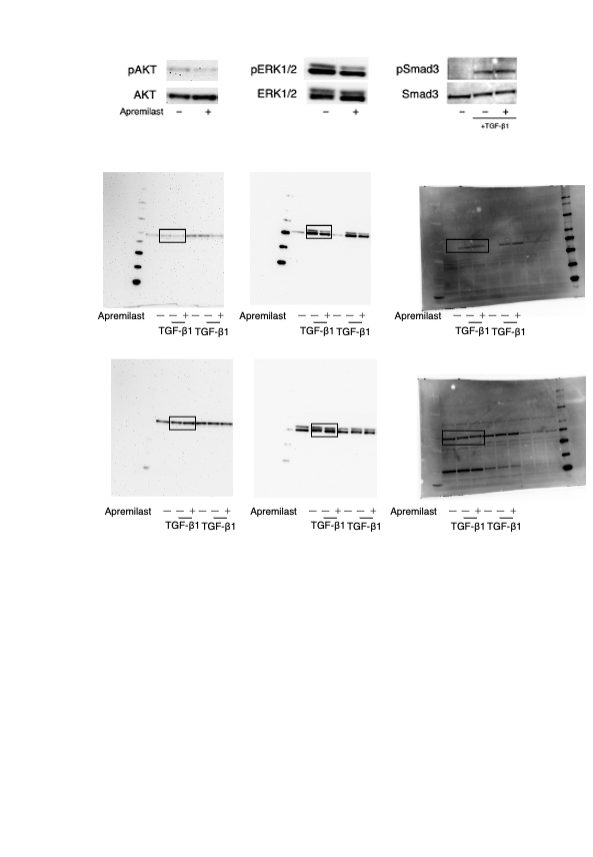


Supplementary Figure 4. Full-length images of Western blotting in Figure 3b.
